# Therapeutic Update in Neonatal Opioid Withdrawal Syndrome: Comparative Effectiveness of Pharmacological Treatments and the ESC Assessment Model. A Systematic Review

**DOI:** 10.1002/hsr2.72289

**Published:** 2026-04-16

**Authors:** José Miguel Pérez‐Jiménez, Marta Periañez Langa, Manuel Coheña Jimenez

**Affiliations:** ^1^ Institute of Biomedicine of Seville (IBiS) Seville Spain; ^2^ Department of Nursing, Schools of Nursing, Physiotherapy and Podiatry University of Seville Seville Spain; ^3^ Research Group CTS1149: Integral and Sustainable Health: Bio‐Psycho‐Social Cultural, and Spiritual Approach for Human Development Seville Spain; ^4^ Human Resources Supervisor and Research Unit Lead Unit University Hospital Virgen Macarena Sevilla Spain; ^5^ Department of Podiatry, Schools of Nursing, Physiotherapy and Podiatry University of Seville, Research Group CTS589: Advances in podiatric surgery Seville Spain

**Keywords:** breast feeding, buprenorphine, clonidine, morphine, neonatal abstinence syndrome, patient care planning

## Abstract

The number of newborns with neonatal opioid withdrawal syndrome is alarming, due to the increase in maternal opioid use. Despite the critical health and socio‐economic impact, a precise clinical and therapeutic approach for opioid using infants and mothers remains undefined.

**Aim:**

The main objective is to update the therapeutic approach and the role of nursing in neonates with neonatal opioid withdrawal syndrome due to mothers who use opioids.

**Design:**

We conducted a systematic review, as a meta‐analysis was not feasible due to heterogeneity across study designs and outcomes, in accordance with PRISMA guidelines and the Cochrane Handbook.

**Methods:**

Studies related to the update of the therapeutic approach and the role of nursing in a newborn with neonatal opioid withdrawal syndrome were considered. Single patient case studies, studies of psychoactive substances other than opium derivatives, iatrogenic treatment approach, guidelines, consensus and meeting abstracts were excluded. The databases Scopus, Dialnet, Cinahl and PubMed were searched.

**Results:**

The results of the 28 publications included indicated the need for a major update of both treatment, diagnostic scales and treatment in neonatal opioid withdrawal syndrome.

**Conclusions:**

Clonidine and buprenorphine demonstrated superior safety profiles and shorter treatment duration compared to morphine in controlling NOWS symptoms. The ESC functional assessment model, integrated with non‐pharmacological interventions, proved superior to traditional Finnegan scoring, significantly reducing pharmacotherapy requirements, total morphine exposure, hospital length of stay, and healthcare costs whilst enhancing breastfeeding rates and parental involvement. Nursing‐led holistic care, encompassing skin‐to‐skin contact, breastfeeding support, rooming‐in, and environmental control, is essential and irreplaceable in comprehensive NOWS management.

## 1 Introduction

Neonatal Opioid Withdrawal Syndrome is understood as the set of signs and symptoms experienced by a newborn when exposure to substances, drugs or narcotics that the mother has consumed during gestation is withdrawn at the time of birth [1]. Based on the above, this paper will focus exclusively on the neonatal abstinence syndrome produced by maternal consumption of opium derivatives.

Neonatal opioid withdrawal syndrome has become a global public health concern, with increasing incidence reported not only in North America but also in Europe, Oceania and several low‐ and middle‐income countries. Recent international studies indicate that the prevalence of NOWS varies widely across regions, reflecting differences in opioid prescribing practices, substance use patterns and access to maternal health care. In Europe, population‐based studies have reported rising rates of NOWS, particularly in countries with increased opioid prescription and opioid use disorder among women of reproductive age. Similarly, data from Australia and New Zealand show a steady increase in hospital admissions related to NOWS over the last decade.

In this context, and despite the growing impact of NOWS, the health emergency and the socio‐economic crisis that opioid use is causing, the clinical and therapeutic approach in newborns opioid exposed and opioid who using mothers has not been precisely determined [2]. Opioid use constitutes not only a public health problem but also a large‐scale socioeconomic crisis. The sustained increase in opioid consumption has been associated with higher mortality rates, overburdened healthcare systems, and substantial economic costs due to lost labor productivity and increased social service needs. In the United States alone, the illicit opioid epidemic was estimated to cost the economy approximately $2.7 trillion in 2023, encompassing losses from deaths, reduced quality of life, healthcare expenses, and crime‐related costs, highlighting how opioid misuse contributes to broader social and economic harm beyond clinical outcomes [3].

Although epidemiological data from low‐ and middle‐income countries are more limited, available evidence suggests that N0WS is likely underdiagnosed and underreported in these settings, due to the lack of standardized diagnostic criteria and surveillance systems. Nevertheless, the growing global burden of opioid use disorder among pregnant women indicates that NOWS represents a significant and emerging worldwide health challenge [4].

When opioids are introduced exogenously and in an uncontrolled manner, the persons stops producing its own, so that the presence of opioids becomes directly dependent on consumption. When opioid levels decrease, the central nervous system senses this. Specifically, it is the locus ceruleus, located in the brain stem, which is responsible for triggering the sympathetic response to situations of panic, stress, anxiety, pain, etc., causing the secretion of norepinephrine at the renal level and the appearance of withdrawal symptoms [5]. This anatomical region controls states of alertness, and is particularly sensitive to opioids. Therefore, its activation affects the normal functioning of the organism. As a consequence, the symptomatology of NOWS is so broad and encompasses these systems: tremors, irritability with excessive or high‐ pitched crying, problems falling asleep, nasal congestion and/or sneezing, low sucking ability, dehydration, loose stools, hyperhidrosis, and seizures [6]. The most common symptom is irritability and crying, which is present in 71.4% of cases [7].

On the other hand, exposure to exogenous opioid substances produces abnormalities in the physiology of the placenta: it decreases the secretion of the enzyme aromatase and increases the expression of enriched placental genes [8], which reduces the amount of nutrients it provides to the foetus (maternal genes and foetal genes that regulate the feeding process must be in balance) [9]. This alteration in the development and growth of the placenta means an increased risk of complications such as miscarriage, premature detachment, amnionitis, placental insufficiency, pre‐ eclampsia, eclampsia, uterine hypertonia, and/or HIV infection, especially through intravenous administration of heroin. As a result, neonates with NOWS are associated with reduced foetal growth, low birth weight and high rates of stillbirth (death at gestational age greater than or equal to 28 weeks, during or after delivery) [10].

In Spain, the Spanish Association of Pediatrics (SAP), describes that the presence of malformations in the ciliated epithelium of the bronchi in newborns born to pregnant women who use opioids causes early chronic respiratory distress [11]. In 72.5% of cases, it is a syndrome with mild symptoms [7].

In addition, opioids are characterised not only by their analgesic and sedative potency, but also by the high speed at which they pass through the placenta by passive diffusion. Symptomatology of a neonate with NOWS does not become evident until the first 48 to 72 h postpartum, and can last from 8 to 16 weeks, depending on the frequency of use, half‐life and dose of opioid consumed. Subacute symptomatology can last up to 6 months, and is related to maternal who use methadone, chlordiazepoxide or methylphenidate. Due to the breadth and generality of the symptoms, differential diagnosis is essential and other pathologies such as hypothyroidism, septic conditions, hypoglycaemia, hypocalcaemia, neurological injury or involvement must be ruled out [12]. Among the most common complications of a newborn exposed to opioids during gestation is the development of postnatal abstinence syndrome [13].

Similarly, there are also health complications for pregnant women, mainly in their mental health, including postpartum depression, anxiety, and/or feelings of guilt, which can lead to suicidal behaviour, especially among younger women [9], as indicated in the study by Margerison et al. [14] with a prevalence of suicide among pregnant mothers who use drugs of 5.4%. As a consequence of the deterioration of maternal mental health, the mother child dyad is directly affected; activities such as breastfeeding (which benefits the newborn by providing nutrients and immunity, if not contraindicated by maternal HIV infection) are reduced or, in most cases, interrupted. But it is not only mental health that is affected; physical symptoms include agitation, rhinorrhoea, abdominal pain, muscle and uterine cramps, myalgia, characteristic of withdrawal. Overdose is the fatal complication of this condition, causing pulmonary oedema, respiratory arrest and coma. Since heroin is the most widely consumed opioid and is administered parenterally, the associated risk is HIV infection, with seropositivity rates of 60‐70% among chronic heroin users, as well as other infectious processes such as hepatitis, sepsis or endocarditis [15, 16]. Therefore, when a gestational process occurs in a woman who consumes opium derivatives, it is considered high‐risk, due to the scarcity and even lack of education on prenatal and postnatal care, the risk of infections associated with consumption practices and the poor nutritional state of the pregnant woman [7]. The main maternal and neonatal complications arising from perinatal opioid exposure, including obstetric complications, neonatal growth restriction, and withdrawal manifestations, are summarised in Table 1.

**Table 1:** Maternal and neonatal complications due to the effect of opioid use.

**Complications in Pregnant Women**	Overdose: respiratory arrest, acute pulmonary edema, coma. Infectious processes through the employed administration route: HIV (60%–70%), endocarditis, hepatitis, sepsis, abscesses. Obstetric complications (miscarriage, premature placental detachment, placental insufficiency, preeclampsia, eclampsia, amnionitis, uterine hypertension). Alterations in cognitive functions, affective disorders, and psychiatric symptoms (anxiety, depression). Symptoms of agitation, abdominal pain, rhinorrhea, irritability, cramps, myalgias (withdrawal syndrome). Vitamin deficiencies and anemias due to folic acid deficiency. Negative impact on the social and family sphere.
**Complications in The Newborn**	Neonatal Opioid Withdrawal Syndrome (**NOWS**). **Obstetric** complications (reduced fetal growth, high stillbirth rates, risk of miscarriage, prematurity). **Congenital malformations.** Risk of sudden infant death syndrome (**SIDS**). **Hypoxia** due to increased oxygen consumption from maternal muscular activity. Long‐term complications in the acquisition of **cognitive, behavioral, immunological, and motor skills.** **HIV** transmission (vertical transmission). Negative impact on the mother‐child dyad.

*Note:* (Amin, A. A. A., et al., 2022; Bada, H. S., et al., 2015; Hudak, M. L. & Tan, R. C., et al., 2012; Maqsood, I., Qazi, G., & Hamayun, Z., 2023).

Currently, the Finnegan or FNASS scale (Finnegan Neonatal Abstinence Scoring Systeme) is used to establish the diagnosis and severity of NOWS, which provides cut‐off points based on the duration and type of symptoms (twenty‐one in total) for initiating pharmacological treatment. Thus, the maximum possible score on the FNASS is 21 points. If a score of 0–7 is obtained, there is no withdrawal syndrome; if it is 8–12, there is a *mild or moderate withdrawal syndrome*; if it is 13–16, it corresponds to *moderate‐severe withdrawal syndrome* and, in the case of a score greater than/equal to 16, *severe withdrawal syndrome* would be diagnosed. A score greater than or equal to 8 in three consecutive assessments implies the start of treatment with morphine [17, 18]. Following the algorithm of application of this diagnostic scale, the first‐line treatment are morphine, methadone and buprenorphine. Second‐line drugs are phenobarbital, clonidine, benzodiazepines and dexmetomidine [18]. This algorithm is developed by the SAP, and indicates the administration of tincture of opium, paregoric elixir or 0.04% sucrose and morphine solution for symptom control. If the pregnant woman has poly‐drug addictive behaviour or uses drugs other than opioids, phenobarbital is administered. Thus, the first‐line drug remains morphine [11]. A comprehensive overview of the pharmacological benefits and associated neonatal risks of opioid‐based treatments currently used in NOWS management is provided in Table 2.

**Table 2:** Neonatal Benefits and Risks Associated with Opioids.

Fármaco	Beneficio neonatal	Riesgo neonatal
**MORPHINE** *(strong agonist with long half‐life)*	Greater control of symptoms With phenobarbital as an adjuvant, shorter LOS and LOT than clonidine, and as monotherapy	High incidence of Neonatal Opioid Withdrawal Syndrome (NOWS) development
**METHADONE** *(strong agonist with long half‐life)*	LOS and LOT shorter than morphine (14%)	Higher rate of readmission to NICU due to the need for adjuvant drugs. Prematurity, low birth weight, high incidence of NOWS, leucodystrophy
**BUPRENORPHINE** *(partial agonist with long half‐life)*	In combination with naloxone, lower risk of NOWS (19% required pharmacotherapy for NOWS) As monotherapy, shorter duration of pharmacological treatment	As monotherapy, an incidence of 42% in the development of NOWS
**CLONIDINE** *(non‐opioid, α‐2 adrenergic agonist)*	Reduces neuronal death (neuroprotective effect) Shorter LOS and LOT than morphine (28 < 39 days on average) Lower risk of NOWS than the rest	Does not reduce risks associated with long‐term opioid exposure

*Note:* (Schiff, D. M. & Grossman, M. R. 2019; Zimmermann, U., et al., 2020).

The ESC (Eat, Sleep, Console) scale is also an alternative clinical tool for the assessment of NOWS, but it focuses on the functional status of the newborn rather than on the detailed quantification of signs and symptoms. It evaluates three key criteria: the neonate's ability to feed adequately (Eat), sleep for at least one continuous hour (Sleep), and be consoled within 10 min (Console), preferably using non‐pharmacological measures. In this context, nursing professionals play a central role, as they are primarily responsible for the continuous assessment of feeding, sleep and consolability, the implementation of non‐pharmacological interventions, caregiver education and family involvement, and the early identification of clinical deterioration requiring medical intervention. Schiff, Grossman [2].

Accordingly, the main objective of this review is to update the therapeutic approach and to highlight the essential role of nursing in the care of neonates diagnosed with Neonatal Opioid Withdrawal Syndrome due to maternal opioid use. Specifically, this review aims to compare the therapeutic efficacy and safety of the most commonly used pharmacological treatments (morphine, methadone, buprenorphine and clonidine) in controlling NOWS symptoms; to analyse the benefits of the ESC scale and non‐pharmacological interventions on neonatal outcomes; to explore the diagnostic accuracy of emerging objective methods for assessing withdrawal symptoms; and to determine the impact of nursing‐led, holistic care on the comprehensive management of Neonatal Opioid Withdrawal Syndrome.

## 2 Methodology

This systematic review, on the update of the therapeutic approach and the role of nursing in a Neonatal Opioid Withdrawal Syndrome, has been carried out following the Cochrane Handbook [19].

After the study selection phase, a systematic process of data analysis and synthesis was carried out. For each study, a data extraction table was developed, recording the design, sample size, population characteristics, type of intervention, outcome measures (Length of Stay (LOS), Length of Treatment (LOT), need for pharmacotherapy, adverse effects, etc.) and main clinical findings.

A deductive thematic coding was then undertaken, guided by the objectives of the review, grouping the studies into three analytic categories:

1. Pharmacological treatment and comparison of medicines (morphine, methadone, buprenorphine, clonidine);
2. Effectiveness of the ESC scale and non‐pharmacological interventions (breastfeeding, skin‐to‐skin contact, rooming‐in, controlled environment);
3. Diagnostic performance and efficiency of the FNASS and ESC scales.

Within each category, a structured narrative synthesis was conducted, comparing the direction and magnitude of reported effects (reduction of LOS and LOT, decrease in morphine exposure, readmission rates, etc.) and highlighting consistencies and discrepancies between studies. Where several articles evaluated the same intervention, the overall consistency of results and the methodological quality of the studies (randomised designs *vs.* retrospective cohorts) were considered in order to underpin the conclusions of the review.

Owing to the heterogeneity of study designs, interventions and reported outcomes, a quantitative meta‐analysis was not considered appropriate. Instead, a qualitative, thematically organised synthesis of the available evidence was undertaken, in line with the guidance of Higgins and Green (2011). Although no meta‐analysis was performed, due to the heterogeneity of the included studies, the work meets the methodological criteria of a systematic review, with an exhaustive search, explicit selection criteria and a structured synthesis.

Four Health Sciences and multidisciplinary electronic databases were consulted and included: Scopus, Dilanet, CINAHL, Pubmed. Before proceeding with the literature search, an expert in scientific literature search methodology, conducted an initial search for articles related to the topic, identifying the terms and descriptors of interest, and with the aim of defining the final search. Thus, the search strategy with language‐free terms and truncations used was as follows: In Pubmed, Cinahl and in Scopus, the following were used: (“Neonatal Abstinence Syndrome” AND “opioid” AND “analgesics” NOT “alcohol”. In Dialnet, the free terms were (“abstinence syndrome” AND “neonat* OR neonate” AND (“opioid* OR opioids”).

### 2.1 Inclusion Criteria

- Publication year between 2015 and 2025. The search period was extended back to 2015 to capture key studies on clonidine and morphine in the treatment of NOWS, as well as foundational research on the use of skin conductance (via sweat measurement) as a quantifiable marker of neonatal stress for early detection and severity assessment of NOWS areas that have not been systematically reviewed to date.
- Full text availability. Only studies available in full text were included to allow complete methodological appraisal and data extraction.
- Study population consisting exclusively of breastfed infants up to 6 months of age. This criterion ensures homogeneity of the sample, given that breastfeeding status and age significantly influence both the pharmacokinetics of opioids and the clinical presentation of NOWS.

### 2.2 Exclusion Criteria

- Single‐patient case reports. Studies reporting only one clinical case were excluded due to their limited generalizability and insufficient statistical basis for drawing conclusions applicable to the broader NOWS population.
- Withdrawal syndromes caused by substances other than opioid derivatives (e.g., alcohol, nicotine, amphetamines, or caffeine). These conditions involve distinct pathophysiological mechanisms, clinical presentations, and management approaches that fall outside the scope of this review, which focuses specifically on opioid‐related NOWS.
- Studies focused on Iatrogenic Withdrawal Syndrome (IWS). Although IWS shares certain clinical features with NOWS, it arises in a fundamentally different context prolonged medical administration of opioids or sedatives in the neonatal intensive care unit and therefore requires a separate therapeutic and diagnostic framework not addressed in this review.

The study selection process was carried out in four phases: identification, screening, selection and inclusion. The literature was reviewed and duplicate papers were removed. Studies were filtered according to title and inclusion criteria. Peer review of abstracts was performed and finally full‐text qualified studies were included.

## 3 Results

**Figure 1:**
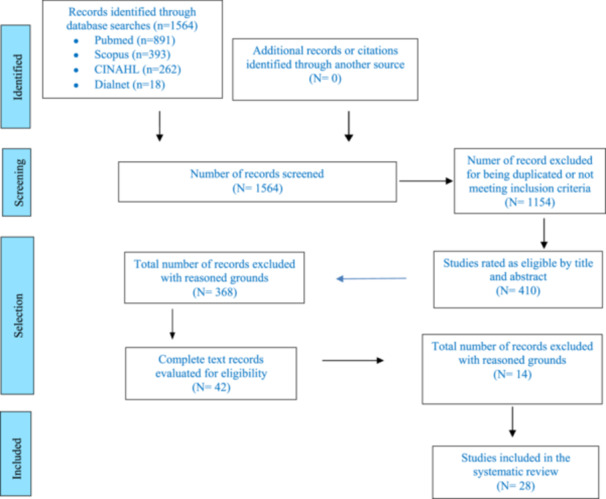
Flowchart of the selection process.

The review process is outlined in the flow chart shown in Figure 1, which presents the study selection according to the PRISMA recommendations. The following is a descriptive summary of the included studies.

The literature search identified 1.564 records in the PubMed, Scopus, CINAHL, and Dialnet databases. After removing duplicates and applying the inclusion and exclusion criteria, 28 studies were included in the review, focusing on pharmacological treatment, non‐pharmacological interventions, and diagnostic tools in Neonatal Opioid Withdrawal Syndrome (NOWS).

As summarised in Table 3 (Summary of data), the evidence was organised into three main categories: pharmacological treatment, non‐pharmacological interventions and ESC based care, and diagnostic tools and assessment methods.

Fourteen studies evaluated pharmacological approaches for the management of Neonatal Opioid Withdrawal Syndrome, primarily comparing morphine, methadone, buprenorphine and clonidine. These studies included retrospective cohort studies and clinical trials and mainly assessed outcomes such as treatment duration, length of hospital stay, safety profiles and readmission rates.

Nine studies focused on non‐pharmacological interventions and care models based on the Eat, Sleep, Console (ESC) approach. These studies, largely observational and quasi‐experimental in design, reported outcomes related to the need for pharmacological treatment, total opioid exposure, length of hospital stay, breastfeeding rates, parental involvement and healthcare costs.

Five studies addressed diagnostic tools and assessment methods for Neonatal Opioid Withdrawal Syndrome, including comparisons between the Finnegan Neonatal Abstinence Scoring System and ESC, toxicological screening methods, and emerging approaches such as cry acoustics and skin conductance. These studies primarily evaluated diagnostic accuracy, feasibility and clinical utility.

With regard to pharmacological treatment, most studies compared the efficacy and safety of morphine, methadone, buprenorphine, and clonidine. Methadone, compared to morphine, was found to be associated with a reduction in treatment duration and hospital stay in some studies, while others found no clear differences and reported higher rates of readmission to the NICU with methadone [20, 21, 22]. Maternal exposure to potent, longacting opioid agonists (morphine, buprenorphine, fentanyl, methadone, oxycodone) in the third trimester was associated with an increased risk of developing NOWS [23]. Clonidine showed a better safety profile and shorter treatment time compared to morphine in controlling NOWS symptoms [21].

With regard to the non‐pharmacological approach and the ESC scale, various studies have shown that its implementation significantly reduces the need for pharmacological treatment, total exposure to morphine, length of hospital stay and costs, while increasing breastfeeding and parental involvement [24, 25, 26, 27, 28]. The combination of the ESC scale with measures such as skin‐to‐skin contact, increased feeding frequency, and rooming‐in was associated with better neonatal outcomes [28, 29, 30, 31].

Anothers studies provided reductions in mean hospital stay by 5.94 days, with no readmissions or complications requiring ICU, by implementing ESC in the therapeutic approach to neonates with NOWS. Consequently, the mean variable cost per patient was decreased by 48% and total neonatal morphine exposure by 79% (from 2.25 mg/kg to 0.45 mg/kg) [24]. Similar conclusions were drawn by Chyi et al. [32] in a retrospective cohort study, where 20.7% of neonates treated with FNASS required pharmacotherapy, while in the other group the percentage was 16.3%. In‐ hospital length of stay was similar in both groups (14.4 days with FNASS and 14.0 days with ESC). These data they pointed out that, although the ESC tool decreases postpartum opioid exposure, the use of this scale independently of FNASS did not result in a significant reduction of in‐hospital stay [30, 32]. In contrast, the study by Blount et al. with 40 infants provided more significant data, observing that the mean length of hospital stay was reduced from 10.3 days with the Finnegan method to 4.9 days with ESC. The need to start morphine drug therapy decreased from 92% with Finnegan to 19% with ESC. There were no readmissions to the NICU. In their trial they explain that all patients were transferred to the mother and child units from the time of diagnosis and initial drug therapy was given as morphine as required by the patients' ESC scores [33].

## 4 Discussion

This review aimed to analyse current evidence on the diagnosis and management of neonatal opioid withdrawal syndrome (NOWS), with particular emphasis on pharmacological strategies, emerging diagnostic approaches, and the role of non‐pharmacological interventions in reducing neonatal stress and improving clinical outcomes. The findings highlight substantial heterogeneity in therapeutic approaches, alongside a growing shift towards care models that prioritise functional assessment and family centred, non‐pharmacological care.

Regarding pharmacological management, morphine remains the most commonly used opioid due to its strong agonist properties. However, available evidence indicates no significant differences in treatment duration or length of hospital stay when morphine is compared with methadone, although morphine may be associated with a greater need for adjunctive medication. In contrast, pooled analyses and recent cohort studies suggest that buprenorphine may be associated with shorter treatment duration and reduced hospitalisation when compared with morphine, positioning it as a potentially favourable first‐line agent for selected infants with NOWS [34, 35].

Morphine and methadone protocols continue to present a clinical dilemma. Pilot randomized data suggest both are reasonable options, but methadone may reduce primary treatment failure and the need for adjunct therapy compared to morphine [36].

Buprenorphine has emerged in recent cohort studies as associated with shorter durations of treatment and hospitalization relative to morphine, corroborating single‐center evidence suggesting it may be a superior first‐line agent for NOWS outcomes [37].

Adjuvant agents such as clonidine and phenobarbital remain widely discussed; reviews note that while clonidine may be used as an opioid‐sparing strategy, data on its independent effectiveness are limited and have mixed outcomes regarding adjunct use and treatment trajectory [38].

Beyond standard pharmacotherapy, emerging pharmacokinetic driven insights emphasize high interpatient variability in drug metabolism among neonates, suggesting that individualized dosing strategies based on pharmacogenetics and developmental pharmacokinetic/pharmacodynamic (PK/PD) models could optimise NOWS treatment beyond traditional score‐based approaches [39].

Other studies have quantified distress and/or stress in neonates, manifested by crying, and caused by NOWS. They established the relationship between electrical activity of the skin and stress. In this way, they showed that high levels of emotional sweating or electrical activity of the skin were related to greater sensitivity to stress and pain. Infants diagnosed with NOWS had higher skin conductance compared to healthy infants [40]. This approach to the importance of skin‐to‐skin therapy for newborns was corroborated by Pérez‐Jiménez et al. in 2023. The authors of this randomised clinical trial demonstrated a reduction in maternal pain of up to 80%, and a reduction in newborn crying, as well as greater success in initiation and establishment of breastfeeding [31].

This variable of crying has been incorporated in other studies, where in addition to objective diagnostic methods based on the basic functional capacities of the newborn, such as eating, sleeping and being comforted, they have included the acoustics of crying. Proponents demonstrated that these acoustics had a high diagnostic accuracy using an ROC curve, with an area under the curve of 0.90, a sensitivity of 0.89 and a specificity of 0.83 [41].

In this respect, the COVID‐19 pandemic has also meant a change in the treatment, care and outcome of infants diagnosed with NOWS, as analysed in the study by MacMillan et al. who concluded that pharmacological monotherapy and the consequent paucity of non‐pharmacological interventions, such as behavioural, environmental and nutritional care, meant higher LOS, LOT and an increased rate of drug treatment failure [29].

However, the most relevant contribution of this study lies in the prioritisation of non‐pharmacological interventions according to their demonstrated effectiveness in reducing NOWS related stress, highlighting parental presence, rooming‐in, and skin‐to‐skin contact as key strategies. These findings are consistent with external evidence suggesting that interventions promoting continuous caregiver–infant interaction are associated with reduced physiological stress responses in neonates, potentially mediated by changes in autonomic regulation and skin conductance activity [40, 42].

Regarding diagnostic assessment, the Finnegan Neonatal Abstinence Scoring System presents important limitations related to its assessment methodology, particularly the requirement for frequent stimulation of the neonate and its emphasis on symptom quantification rather than functional status. These factors may lead to longer Neonatal Intensive Care Unit stays and increased healthcare costs. In contrast, the Eat, Sleep, Console (ESC) care model, which emphasises function‐based assessment and enhanced non‐pharmacological interventions, has been shown to significantly reduce the need for pharmacotherapy and to shorten the time to readiness for discharge when compared with traditional scoring systems, as demonstrated in controlled multicentre studies [43, 44].

Metaanalytic evidence across multiple studies further reinforces that ESC not only reduces pharmacotherapy requirements but also significantly decreases hospital length of stay and opioid treatment duration compared to Finnegan scoring approaches [45].

Conversely, some retrospective analyses suggest that broader improvements in outcomes over time may not be solely attributable to differences in the assessment tool itself, indicating that evolving care practices including environmental and feeding support may partly drive observed benefits [46].

Weight change studies comparing ESC and Finnegan models show no evidence of excessive early postnatal weight loss with ESC, suggesting the safety of ESC with respect to neonatal growth metrics, which supports its application alongside prioritized breastfeeding support [47].

External comparative research also highlights that while ESC reduces initial pharmacotherapy initiation rates, continuous evaluation of nutrition support and lactation support is critical during transitions between scoring models to optimize neonatal outcomes [34].

Overall, the integration of these findings underscores a converging trend towards strategies that prioritise non‐pharmacological care and more nuanced pharmacotherapeutic selection particularly the use of buprenorphine and ESC‐based care models to improve outcomes for infants with NOWS [43, 44, 45].

Some studies point out that the most effective cytotoxic tests for the diagnosis of NOWS are focused on monitoring maternal consumption during gestation, by means of blood, oral secretions and sweat analysis [48]. However, other authors object to having a very narrow detection window, since opioids in the body have a half‐life of 3 days, as is the case with heroin, which has a half‐life of 72 h (Hudak & Tan, 2012). They are also primarily focused on detecting alcohol use, which is the most studied drug with the most standardised tests.

The update of the protocol for analgesia in withdrawal syndrome in the Paediatric Intensive Care Unit published by the SAP in 2020 establishes clonidine, administered orally, as the firstline drug, and phenobarbital, in case of impossibility of oral administration and/or severe NOWS, with administration of rescue boluses of morphine in case of severe agitation (SAP, 2023).

A critical consideration in the comprehensive management of NOWS relates to the clinical frameworks that guide assessment and intervention. The Finnegan scale, while widely adopted, has inherent limitations in its clinical application framework, as it primarily drives a pharmacotherapy‐centered approach without systematically integrating behavioural, environmental, and nutritional interventions into the therapeutic protocol [49]. This symptom focused scoring methodology may inadvertently narrow the scope of nursing care plans for NOWS management, potentially limiting clinical attention to pharmacological interventions while underutilizing the holistic and comprehensive care strategies that are central to contemporary neonatal nursing practice [02].

In contrast, the ESC (Eat, Sleep, Console) assessment model represents a paradigm shift toward function‐based, family‐integrated care. By prioritising the neonate's ability to feed adequately, achieve consolidated sleep, and be consoled through caregiving interventions, the ESC framework inherently emphasizes non‐pharmacological strategies as the foundation of care. This approach not only reduces reliance on pharmacological treatment and shortens hospital length of stay, but also recenters clinical practice around the infant's functional well‐being and the parent‐infant dyad. The ESC model thereby promotes a more holistic and humanised approach to neonatal care, addressing key limitations of the Finnegan scale by expanding the therapeutic focus beyond symptom quantification to encompass environmental modification, nutritional optimization, and family engagement as core components of NOWS management.

## 5 Conclusions

This systematic review evaluated the evidence published between 2015 and 2025 to update the therapeutic approach and define the role of nursing in neonates with neonatal opioid withdrawal syndrome (NOWS) due to maternal opioid use. The principal findings in relation to the stated objectives are as follows:

− Pharmacological treatment efficacy and safety: Comparison of the most commonly used pharmacological treatments (morphine, methadone, buprenorphine and clonidine) reveals that clonidine demonstrates superior safety profiles and shorter treatment duration compared to morphine in controlling NOWS symptoms. Phenobarbital as adjuvant therapy also shows efficacy, particularly in severe cases. Buprenorphine emerges as a promising first‐line agent with reduced treatment and hospitalization durations compared to traditional opioid agonists.
− Benefits of the ESC scale and non‐pharmacological interventions**:** The Eat, Sleep, Console (ESC) approach, integrated with non‐pharmacological interventions including breastfeeding support, skin‐to‐skin contact, rooming‐in, and controlled environmental measures, significantly improves neonatal outcomes. Implementation results in substantially reduced need for pharmacotherapy, shortened hospital stays, decreased total morphine exposure, lower healthcare costs, and enhanced rates of breastfeeding and parental involvement.
− Diagnostic accuracy of emerging assessment methods: While the Finnegan Neonatal Abstinence Scoring System (FNASS) and ESC demonstrate comparable diagnostic sensitivity and specificity, the ESC model provides superior functional assessment and therapeutic guidance. Emerging objective diagnostic methods, including cry acoustics analysis and skin conductance measurement, offer promising adjunctive diagnostic tools for early detection and severity assessment of NOWS.
− Impact of nursing‐led holistic care: Nursing professionals play a central and irreplaceable role in NOWS management through the ESC framework. The nursing care plan encompasses comprehensive assessment of feeding capacity, sleep patterns, and consolability; implementation of non‐pharmacological interventions including skin care, tactile stimulation, frequent feeding schedules, and skin‐to‐skin contact; family education and support for maternal‐infant bonding; and early identification of clinical deterioration requiring medical intervention. The reduction of non‐pharmacological nursing interventions, as observed during the COVID‐19 pandemic, resulted in increased treatment duration, prolonged hospital stays, and higher rates of pharmacological treatment failure, underscoring the critical importance of nursing involvement.
− Integrated care model recommendation**:** A complementary diagnostic approach utilizing both Finnegan and ESC scales for initial assessment, combined with ESC‐guided therapeutic management emphasizing non‐pharmacological interventions, represents the optimal evidence‐based strategy for NOWS care.

In conclusion, current evidence supports the integration of clonidine or phenobarbital based pharmacotherapy, the ESC functional assessment model, and structured nursing led non‐pharmacological interventions as the cornerstone of comprehensive, familycentered management of neonatal opioid withdrawal syndrome.

## 6 Relevance to Clinical Practice

Due to the great impact generated by neonatal opioid withdrawal syndrome (NOWS), it is necessary to update the diagnostic scales and the most commonly used treatments to control the symptomatology of this disease. The identification of clonidine as the most effective and safe drug for the control of the symptomatology of this syndrome represents a change in the current treatment and it is important that nurses become familiar and skilled in its administration and monitoring. The role of nursing is crucial in the therapeutic approach to the neonate with NOWS. The reduction of non‐pharmacological interventions resulted in a significant increase in the duration of treatment, hospital stay and drug treatment failure rate.

## Author Contributions

**José Miguel Pérez‐JIménez:** conceptualization, data curation, formal analysis, investigation, methodology, resources, software, supervision, validation, visualization, writing – original draft, writing – review and editing. **Marta Periañez Langa:** conceptualization, formal analysis, investigation, methodology, visualization, writing – original draft, writing – review and editing. **Manuel Coheña Jimenez:** conceptualization, data curation, formal analysis, investigation, methodology, resources, visualization, writing – original draft, writing – review and editing.

## Funding

The authors received no specific funding for this work.

## Conflicts of Interest

The authors declare no conflicts of interest.

## 1 Transparency Statement

The lead author José Miguel PÉREZ‐JIMÉNEZ affirms that this manuscript is an honest, accurate, and transparent account of the study being reported; that no important aspects of the study have been omitted; and that any discrepancies from the study as planned (and, if relevant, registered) have been explained.

## Supporting information

Supporting File 1

Supporting File 2

## Data Availability

The data that support the findings of this study are available from the corresponding author upon reasonable request.
