# Supplementary material for: Therapeutic Update in Neonatal Opioid Withdrawal Syndrome: Comparative Effectiveness of Pharmacological Treatments and the ESC Assessment Model. A Systematic Review
Source: Health Sci Rep. 2026 Apr 16;9(4):e72289. doi: 10.1002/hsr2.72289 (PMC13086615; doi:10.1002/hsr2.72289)
Supplement: Supplementary file 2 — Supporting File 2 [file HSR2-9-e72289-s002.docx]

TABLE 3 . Summary of data

| **Author and year** | **Type of study** | **Sample** | **Target** | **Results** | **Variable** |
| --- | --- | --- | --- | --- | --- |
| Zimmermann et al. (2020) | Multicentre, double-blind, parallel-group, multicentre study  with three arms (2001-  2007) | Neonates from seven NICU units of opioid- addicted mothers in Zurich.  N=143 neonates | To compare the efficacy of morphine, chlorpromazine and phenobarbital in the control and treatment of NOWS. | The length of stay or treatment was similar for all three opioids. However, the use of morphine alone or in combination decreases the duration of treatment, as well as the duration of treatment.  as morphine alone controls the symptomatology better. | Pharmacological line of treatment. |
| Sutter et al. (2022) | Randomised controlled trial (2016- 2018) | Infants with prenatal opioid exposure and gestation greater than or equal to 34 weeks (excluding buprenorphine cases) N=61 infants. | To analyse the length of hospital stay (LOS) and length of treatment (LOT) of NOWS with methadone and with morphine. | There are no significant differences between LOS and LOT, but those treated with methadone had a higher rate of readmission to the NICU due to excessive sedation as they required more sedation. doses of morphine-like opioids to control symptoms. | Pharmacological line of treatment. |
| Esposito et al. (2022) | Study of cohorts (2000-  2014) | Pregnant women exposed to opioid analgesics in the third trimester of pregnancy.  N= 48202 pregnant women. | To compare the risk of developing NOWS by analgesic monotherapy in the third trimester of pregnancy. | Strong agonists with a long half-life pose a higher risk of developing NOWS (morphine, buprenorphine, fentanyl methadone, oxycodone). | Pharmacological line of treatment. |


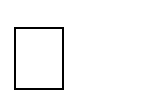


| Mullins et al. (2020) | Retrospective cohort study (2014- 2014).  2018) | Pregnant women in treatment for opioid withdrawal syndrome. N= 193 pregnant women. | To know the rate of NOWS produced by withdrawal treatment with buprenorphine monotherapy and with  combined buprenorphine and naloxone therapy. | The combined action of buprenorphine and naloxone meant less risk of developing NOWS. | Pharmacological line of treatment. |
| --- | --- | --- | --- | --- | --- |
| Wang et al. (2023) | Retrospective cohort study (2008- 2008).  2016) | Early (0-20 weeks) and late ( 20 weeks) pregnant women with opioid use disorder. N=152 pregnant women. | To assess the risk of developing NOWS as a function of gestational age with buprenorphine and methadone monotherapy. | Methadone exposure was generally associated with an increased risk of prematurity, NICU admission, low birth weight and low birth weight.  birth weight, development of NOWS, increased LOS and respiratory symptoms. | Pharmacological line of treatment. |
| Maqsood et al. (2023) | Retrospective cohort study (2021- 2021-  2021- 2021-  2021- 2021).  2022) | Neonates with in utero opioid exposure.  N = 180 neonates | To study the safety and efficacy of morphine and methadone for treating NOWS. | The use of methadone to control NOWS resulted in lower LOS and shorter treatment duration. | Pharmacological line of treatment. |
| Davis et al. (2018) | Double-blind, randomised study (2014-  2017) | Neonates at terms of 8 NICU with exposure to buprenorphine, methadone and other opioids during pregnancy.  N= 116 neonates. | To compare LOS and LOT in neonates with NOWS treated with methadone and morphine. | The use of methadone as a pharmacological treatment was associated with a 14% lower LOS and LOT compared to morphine. | Pharmacological line of treatment. |
| Nguyen et al. (2018) | Retrospective cohort study | Pregnant women with opioid addiction treated with buprenorphine and naloxone. N=26 | To know the rate or incidence of NOWS caused by maternal buprenorphine and naloxone treatment. | Treatment with combined buprenorphine and naloxone for the management of maternal withdrawal syndrome has resulted in the birth of babies with normal birth parameters.  Only 19% required drugs to control  NOWS. | Pharmacological line of treatment. |

|  |  | pregnant women |  |  |  |
| --- | --- | --- | --- | --- | --- |
| Kanervo et al. (2023) | Retrospective cohort study | Pregnant women with opioid addiction treated with buprenorphine- naloxone, buprenorphine and methadone. N=172 pregnant women | To compare the impact on newborns of pregnant women in opioid withdrawal treatment with buprenorphine-naloxone, buprenorphine and methadone. | The buprenorphine-naloxone and naloxone groups did not differ significantly in the results. In contrast, polydrug use was more frequent in the methadone group. The smallest neonates were those who had been exposed to methadone. The need for pharmacotherapy to control NOWS was less in the  exposed to buprenorphine. | Pharmacological line of treatment. |
| Brusseau et al. (2020) | Prospective, randomised cohort study. | Term infants with morphine treatment failure, additionally administered clonidine or phenobarbital. N=24 infants. | Know LOS and LOT of adjuvant morphine therapy with phenobarbital and clonidine. | Phenobarbital as an adjuvant to morphine resulted in lower LOS and LOT than adjuvant clonidine and morphine monotherapy. | Pharmacological line of treatment. |
| Grasch et al. (2023) | Retrospective cohort study | Mother-infant dyads with term delivery and treatment for opioid withdrawal syndrome with buprenorphine.  N= 12 mother-child dyads. | To study the incidence of NOWS due to therapeutic consumption of buprenorphine in pregnant women treated for opioid addiction by concentration of metabolites in umbilical cord, amniotic fluid and blood plasma.  maternal | Forty-two per cent of the newborns developed NOWS, which had high concentrations of buprenorphine metabolites in the umbilical cord and amniotic fluid. The concentration of the drug in maternal blood plasma was similar in both groups. | Pharmacological line of treatment. |

| Bada et al. (2015) | Prospective cohort study | Term newborns with prenatal opioid exposure admitted to NICU. N= 31  newborns | Comparing the therapeutic efficacy of clonidine and morphine in NOWS | LOT was higher on morphine than on clonidine (39 days and 28 days on average, respectively). Morphine-treated infants had greater excitability. Cognitive,  neurological and motor development was similar. | Pharmacological line of treatment. |
| --- | --- | --- | --- | --- | --- |
| Mahnke et al. (2022) | Prospective cohort study | Pregnant women under pharmacological treatment for opioid exposure. N= 58 pregnant women. | To study whether the severity of NOWS can be predicted by m- RNA present in the umbilical cord of newborns. | The presence of mRNAs in the umbilical cord predicts the need for drug treatment and prolonged hospitalisation. | Pharmacological line of treatment. |
| Miller, JS, et al., (2024). | Retrospective cohort | Infants diagnosed with NAS n = 182 | Identify specific signs in infants requiring long-term treatment for NAS, | Resurgence of signs with prolonged treatment duration is mainly a consequence of opioid tolerance or withdrawal. | Pharmacological line of treatment. |
| Dodds et al. (2019) | Retrospective, randomised, cohort study. | Neonates with prenatal opioid exposure admitted to NICU for NAS. N=  82 neonates | To study the benefits of implementing the ESC diagnostic and assessment scale. | The ESC approach reduced LOS, with no readmissions to NICUs. It reduced the average cost per patient by 48% and total morphine exposure (use of pharmacological treatment) by 79%. | ESC diagnostic scale and non- pharmacological approach. |
| Andrew et al. (2022) | Prospective controlled cohort study | Term infants exposed and not exposed to opioids during gestation. N=117 infants | To study the possibility of using the ROC curve to take the acoustics of infant crying as a diagnostic variable for NOWS.  Born. | It was concluded that the measurement of the acoustics of crying has a high diagnostic accuracy, with an area under the curve of 0.90, sensitivity of 0.89 and specificity of 0.89.  of 0.83. | ESC diagnostic scale and non- pharmacological approach. |

| Kathryin et al. (2022) | Multicentre retrospective study | Neonates with prenatal opioid exposure born at term in 2019-2020  N= 1139 neonates | To analyse the impact of the pandemic and the consequent loss of non-pharmacological neonatal care on the outcome of neonates with NOWS. | The shortage of non-pharmacological care (and the impossibility of implementing nursing care) meant higher LOS and LOT, as well as a higher rate of drug treatment failure. | ESC diagnostic scale and non- pharmacological approach. |
| --- | --- | --- | --- | --- | --- |
| Favara et al. (2019) | Retrospective cohort study | Newborns with prenatal opioid exposure admitted to NICU. N= 1738  newborns. | To assess whether breast milk- based feeding of infants with NOWS results in lower LOS and LOT compared to formula milk. | Breast milk-based feeding of infants with NOWS decreased LOS and LOT compared to formula feeding. | ESC diagnostic scale and non- pharmacological approach. |
| Michelle et al.(2023) | Retrospective cohort study | Neonates with perinatal opioid exposure, admitted to the NICU for withdrawal syndrome. N=52 neonates. | To evaluate the benefits of the implementation of the ESC diagnostic scale in the evolution of NOWS. | The implementation of the ESC diagnostic tool has resulted in the reduction of the  of morphine doses to control NOWS symptomatology, as well as breastfeeding increased by 19%. | ESC diagnostic scale and non- pharmacological approach. |
| Achilles & Castaneda- Lovato (2019) | Prospective cohort study | Neonates with perinatal opioid exposure admitted to the NICU for NAS. N=181 newborns | Compare LOS and LOT in groups of newborns assessed with FNASS and ESC, respectively | ESC has resulted in a reduced need for methadone administration for symptom control, as well as a decrease in LOS and LOT and lower healthcare costs. | ESC diagnostic scale and non- pharmacological approach. |
| Grossman et al. (2018) | Retrospective cohort study | Perinatally opioid- exposed newborns admitted to NICU. N=50 newborns | To study the efficacy of ESC in the initiation of pharmacological treatment for  NOWS. | The ESC approach reduced drug therapy in newborns with NOWS from 62% to 12%. It also reduced LOS from 16 to 6 days. | ESC diagnostic scale and non- pharmacological approach. |

| Schubach et al. (2016) | Prospective cohort study | 12 infants with NOWS and 12 infants not exposed to opioids in the process gestational age. N= 24 infants in total | To study the potential of skin conductance to understand and quantify stress or distress in the infant with NOWS. | Skin conductance was 49% higher in infants with NOWS than in the control group. | ESC diagnostic scale and non- pharmacological approach. |
| --- | --- | --- | --- | --- | --- |
| Pérez-Jiménez J.M., et al.(2023) | Randomised clinical trial | 40 postpartum caesarean section women with skin-to- skin activity and 40 postpartum caesarean section women without skin- to-skin activity N=  83 women. | To analyse the benefits of skin- to-skin activity on haemoglobin levels and uterine contractions in caesarean deliveries. | Skin-to-skin activity resulted in greater postpartum uterine contractions, lower maternal haemoglobin decline, decreased pain and higher breastfeeding success rate. | ESC diagnostic scale and non- pharmacological approach. |
| Mascarenhas, M., et al.,  (2024). | Retrospectiv. cohort study | Infants diagnosed with NAS n = 182 | Identify specific signs in infants requiring long-term treatment for NAS, | Resurgence of signs with prolonged treatment duration is mainly a consequence of opioid tolerance or withdrawal. | Effectiveness and diagnostic efficiency of the FNASS and ESC  scales. |
| Blount et al. (2019) | Retrospective cohort study | Term infants with gestational opioid exposure admitted to the NICU. N= 40  infants. | To know the benefits in terms of LOS and LOT of applying the ESC diagnostic scale and therapeutic approach, in contrast to the Finnegan scale. | The average length of stay in hospital (ALOS) was reduced from 10.3 days with the Finnegan method to 4.9 days with ESC. The need to start morphine drug therapy decreased from 92% with  Finnegan to 19% with ESC. | Effectiveness and diagnostic efficiency of the FNASS and ESC  scales. |

| Chyi et al. (2022) | Retrospective cohort study | Term infants with perinatal exposure to methadone, buprenorphine, heroin or opioid analgesics. N=264 infants. | To compare the effectiveness of ESC and FNASS assessment tools in diagnosing and monitoring NOWS. | The ESC diagnostic tool alone does not lead to a significant reduction in LOS, LOT or initiation of pharmacotherapy. | Effectiveness and diagnostic efficiency of the FNASS and ESC  scales. |
| --- | --- | --- | --- | --- | --- |
| Amee et al. (2022) | Retrospective cohort study | Neonates with prenatal opioid exposure measured with ESC and FNASS during the first 5 days of life. N= 64 infants. | To compare the efficacy of the ESC and FNASS assessment tools in the diagnosis and control of NOWS and to find out what non-pharmacological measures are available.  greater efficiency. | The assessment tool and the ESC approach used separately to FNASS reduces the use of pharmacological treatment and hospital stay by 7%. | Effectiveness and diagnostic efficiency of the FNASS and ESC  scales. |
| Ryan et al. (2021) | Retrospective cohort study | Neonates with prenatal opioid exposure as measured by M- FNASS and M- FNASS with ESC approach. N=158  neonates | To know the sensitivity and specificity of the ESC scale compared to FNASS. | The ESC approach and combined assessment (M-FNASS and ESC) means lower LOS and reduced use and duration of drug treatment. | Effectiveness and diagnostic efficiency of the FNASS and ESC  scales. |
